# Structure of a Bacterial Virus DNA-Injection Protein Complex Reveals a Decameric Assembly with a Constricted Molecular Channel

**DOI:** 10.1371/journal.pone.0149337

**Published:** 2016-02-16

**Authors:** Haiyan Zhao, Jeffrey A. Speir, Tsutomu Matsui, Zihan Lin, Lingfei Liang, Anna Y. Lynn, Brittany Varnado, Thomas M. Weiss, Liang Tang

**Affiliations:** 1 Department of Molecular Biosciences, University of Kansas, 1200 Sunnyside Avenue, Lawrence, Kansas, United States of America; 2 National Resource for Automated Molecular Microscopy, The Scripps Research Institute, La Jolla, California, United States of America; 3 Stanford Synchrotron Radiation Lightsource, SLAC National Accelerator Laboratory, Stanford University, 14 2575 Sand Hill Road, MS69, Menlo Park, California, United States of America; ContraFect Corporation, UNITED STATES

## Abstract

The multi-layered cell envelope structure of Gram-negative bacteria represents significant physical and chemical barriers for short-tailed phages to inject phage DNA into the host cytoplasm. Here we show that a DNA-injection protein of bacteriophage Sf6, gp12, forms a 465-kDa, decameric assembly *in vitro*. The electron microscopic structure of the gp12 assembly shows a ~150-Å, mushroom-like architecture consisting of a crown domain and a tube-like domain, which embraces a 25-Å-wide channel that could precisely accommodate dsDNA. The constricted channel suggests that gp12 mediates rapid, uni-directional injection of phage DNA into host cells by providing a molecular conduit for DNA translocation. The assembly exhibits a 10-fold symmetry, which may be a common feature among DNA-injection proteins of P22-like phages and may suggest a symmetry mismatch with respect to the 6-fold symmetric phage tail. The gp12 monomer is highly flexible in solution, supporting a mechanism for translocation of the protein through the conduit of the phage tail toward the host cell envelope, where it assembles into a DNA-injection device.

## Introduction

Viruses have evolved a variety of mechanisms to penetrate the host cell envelope and send viral genetic material into the host cell to establish infection and enable production of progeny viruses. Gram-negative bacteria have a sophisticated cell envelope structure consisting of the outer membrane, the peptidoglycan and the inner membrane [1–3], posing a particular challenge to their viruses, as viral nucleic acid must be injected across those three barriers into host cytoplasm without breaching the cell membrane integrity and without being digested by nucleases in the periplasmic space. Myoviruses such as phage T4 use a contractile tail to abruptly pierce a hole in the host cell envelope to allow DNA translocation, which also involves hydrolysis of peptidoglycan through an enzymatic activity associated with one of the protein components in the tail [4–6]. In contrast, members of the podovirus family are characteristic of short, non-contractile tails, which are not long enough to traverse the host cell envelope as evident in structures of phage particles [7, 8]. These short-tailed phages hence require additional “pilot” proteins, or DNA-injection proteins, to guide the phage DNA across host cell envelope into the cytoplasm, presumably by forming a channel traversing the host cell envelope. Studies of podoviruses such as P22 and T7 showed that several phage structural proteins were associated with host cell membrane upon host attachment and were required for translocation of phage DNA into host cytoplasm [9–11]. Electron tomography studies showed that, upon attachment to host cells, T7 formed an elongated structure spanning the host cell envelope, which was thought to be formed by phage internal core proteins [12]. Similar elongated density spanning the host cell envelope was also observed for phage epsilon15 [13], and 40- to 55-nm extension was observed for phage T7 tails penetrating membrane vesicles [14].

How phage DNA-injection proteins form or help form a channel across the host cell envelope is not known, and the exact roles of those DNA-injection proteins in such a process remain largely unclear. In phage P22, four structural proteins, gp7, gp20, gp16 and gp26, were reported to mediate phage DNA injection into hosts [9]. Among those, gp16 was shown to be associated with membrane [15], and gp26 was shown to form a needle-like structure at the distal end of the phage tail and might be dislodged from the phage tail upon host attachment to make the way for DNA injection and also may perturb the host cell membrane or peptidoglycan [9, 16–22]. Phage Sf6 also possesses such a needle-like structure at the tail end [23], and Sf6 gp9, an ortholog to P22 gp26, contains a conserved coiled-coil domain and a knob domain similar to P22 gp26 [24, 25].

Given the short replication cycle of tailed dsDNA bacteriophages, the time span from phage attachment to host cells and injection of phage DNA into the host cytoplasm is brief, which typically completes within a few minutes [26]. How such a uni-directional, rapid DNA injection process is carried out remains unclear. Moreover, in most tailed dsDNA bacteriophages, the dsDNA genome is highly densely packaged at ~500 mg/ml, a density that nears that of the crystalline DNA, as a result of the DNA packaging process conducted by virally encoded motor proteins [27–29]. Such a dense packaging of dsDNA in a confined small volume of the phage capsid is energetically unfavorable, and may play a role in driving DNA from within the phage capsid into the host cells [26, 30].

Sf6 is a tailed dsDNA phage that infects *Shigella flexneri*, and is a close relative to phage P22 [31]. Like other members of the podovirus family, Sf6 possesses a short, non-contractile tail emanating from an icosahedral capsid [23, 32]. Phage Sf6 contains three DNA-injection proteins, gp11, gp12 and gp13, which are homologous to gp7, gp20, and gp16 in P22, respectively [31]. Among them, gp12 is a 431-residue, 46.5kDa protein. Here we show that heterologously expressed Sf6 gp12 assembles into a decamer in vitro. The electron microscopic structure of the gp12 decameric assembly shows a constricted channel, which may provide the conduit for the rapid, uni-directional translocation of phage dsDNA into host cell cytoplasm. The 10-fold symmetry is rarely seen in biological systems but may be a common feature among P22-like phages. Such a 10-fold symmetry suggests a symmetry mismatch against the 6-fold symmetric phage tail. Additionally, solution X-ray scattering shows that gp12 monomer exhibits high flexibility, supporting a mechanism for translocation of this protein from within the phage particle through the tail conduit to the host cell envelope.

## Materials and Methods

### Production of the gp12 decameric assembly

**Fig 1:**
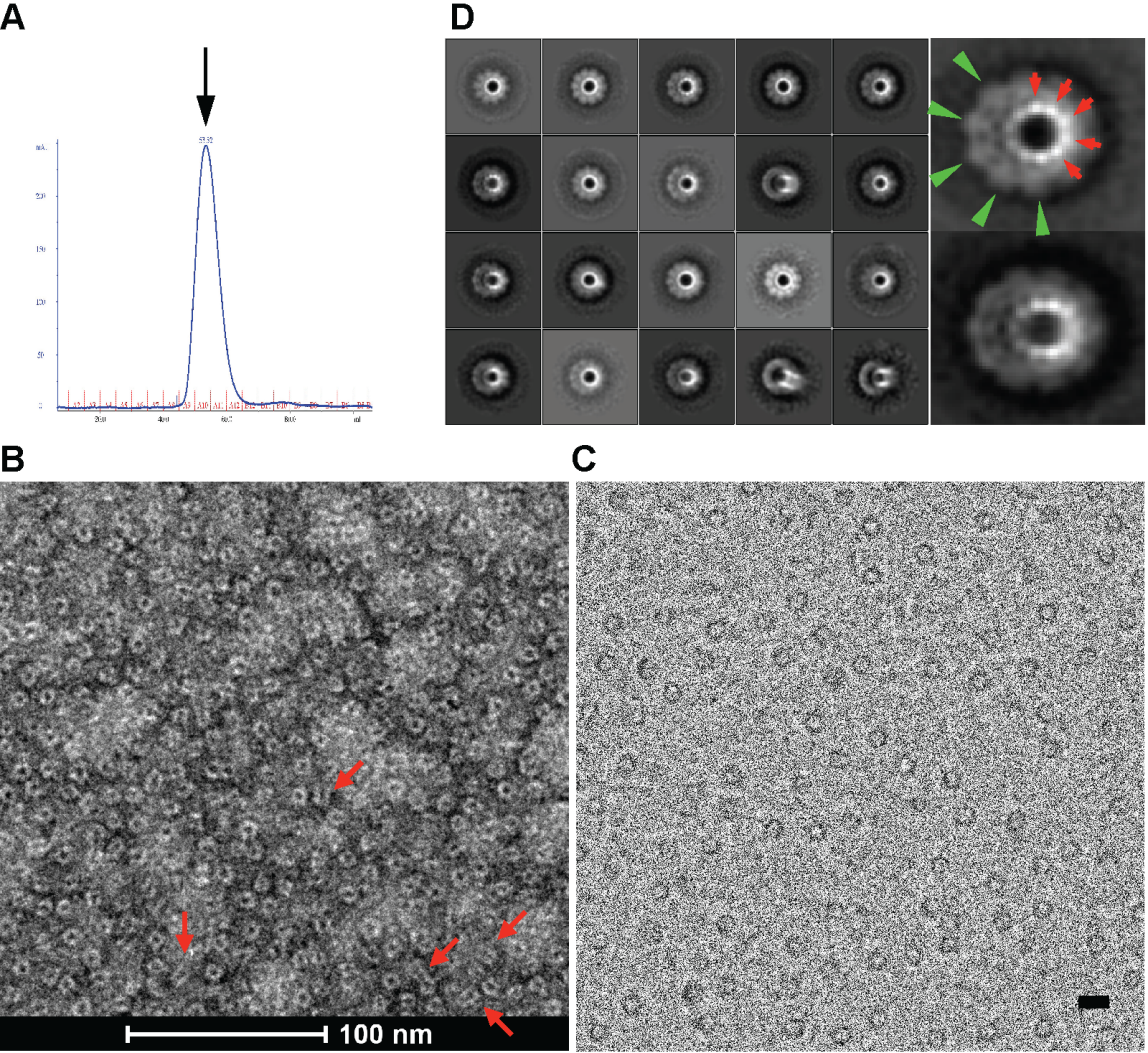
The gp12 assembles into a ring-like decamer. (A) Size exclusion chromatogram of the purified gp12 decamer. The peak corresponds to an estimated molecular weight of 471kDa, indicating a decamer (arrow). (B) Electron micrograph of the purified gp12 decamer negatively stained with uranyl acetate. Notice the dominant views are down the longitudinal axis of the gp12 decamer. Some side or tilted views are indicated with red arrows. (C) Electron micrograph of the frozen-hydrated gp12 decamer. Bar, 100 Å. (D) Class averages of the frozen-hydrated gp12 decamer. Enlarged views of two class averages are shown on the right. The 10-fold symmetry is clearly evident for both the crown (green arrowheads) and the stem domains (red arrows). The box size for class averages in the left panel is 309.8 Å.

The DNA encoding gene *12* in phage Sf6 genome was cloned into vector pET28b. The resultant plasmid containing gene *12* was transformed into *E*. *coli* BL21(DE3) cells. Cultures were grown to an optical density (OD) of 0.5 to 0.7, and expression was induced with 1mM IPTG at 30°C. Cell growth was continued for 4 hours. Cells were harvested by centrifugation at 15,000 rpm on a Sorvall RC6+ Superspeed centrifuge with an SS-34 rotor at 4°C for 1 hour. Supernatant was discarded. Cell pellets were resuspended in buffer A (20mM NaPO4 pH7.4, 100mM NaCl, 1mM EDTA), then centrifuged at 15,000 rpm for 15 minutes using the same rotor as described above. Supernatant was discarded, and pellets were resuspended in buffer A with 8M urea followed by nutation for 16 hours at room temperature to completely denature the proteins. The solution containing the denatured proteins was centrifuged at 15,000 rpm for 1 hour using the same rotor as described above. The debris was discarded and the supernatant was renatured by step dilution with buffer A to 4M, 2M and 1.3M urea and incubation at 4°C for half hour for each dilution step. After that, the solution was dialyzed against buffer A at 4°C for overnight, dialyzed against buffer B (20mM NaPO4 pH7.4) for overnight, then dialyzed against fresh buffer B for 4 hours. The solution containing the renatured proteins was centrifuged at 15,000 rpm for 1 hour using the same rotor as described above, and the supernatant was loaded onto a Ni-NTA column equilibrated with buffer B. The column was washed with buffer B with 20mM imidazole, and eluted with 50mM, 100mM, 200mM and 500 mM imidazole sequentially. The fractions of 200mM imidazole elution were concentrated and loaded on a Hiload Superdex 200 column (GE Healthcare). A peak corresponding to a decamer was typically observed (Fig 1A), but peaks corresponding to a monomer may sometimes be observed (S1B Fig). The peak fractions at 54.6 ml corresponding to the gp12 decamer and at 73.6 ml corresponding to the gp12 monomer were collected and concentrated to 8.3 mg/ml and 5.6 mg/ml respectively for subsequent analysis.

### Electron microscopy and image analysis

The gp12 decamer sample at 8.3 mg/ml was diluted 30-fold immediately prior to adsorption onto an EM grid, and negatively stained with 1% uranyl acetate. The grid was air dried, and examined on an FEI Tecnai F20 transmission electron microscope operating at 200kV. For negative staining EM data collection, the gp12 sample was diluted by 200 fold and stained with 2% uranyl acetate, and 494 micrographs were recorded under the low-dose condition with a total dose of 38.89 electrons/Å^2^ using Leginon [33] on an FEI Tecnai TF20 electron microscope operating at 200 kV with a Tietz TemF416 4k x 4k CMOS camera (TVIPS GmbH) at a 62,000x magnification. A total of 86,783 particles were boxed with the program DoGPicker [34] in the Appion pipeline [35] using boxes with a dimension of 128x128 pixels and a pixel size of 2.73 Å. Contrast transfer function (CTF) correction was performed with the program CTFFind [36], with calculated defocus values ranging from -0.29 to -2.57 μm. Image analysis and 3D reconstruction was performed with RELION [37]. Reference-free 2D classification was performed without applying any symmetry. After 2D classification, 54,104 particles associated with high-quality class averages were included for the subsequent 3D reconstruction. The starting model was generated with the program e2initialmodel.py in EMAN2 [38] using the class averages from 2D classification. The starting model was filtered at 30 Å resolution for gold standard refinement with RELION. In the 3D reconstruction, 10-fold rotational symmetry (as demonstrated in cryoEM image analysis; see below) was applied. The resolution of the 3D reconstruction was estimated to be 13.4 Å using the FSC = 0.5 criterion, using a mask automatically generated by RELION. The handedness of the model was not determined. The robustness of the 3D reconstruction was also confirmed by using the stating model low-pass filtered at 60 Å resolution, which gave rise to an essentially identical 3D reconstruction. The EM map has been deposited with the EMDataBank with the accession code EMD-6330.

For cryoEM, the gp12 sample at 8.3mg/ml was diluted by 5 fold and frozen hydrated. CryoEM data were collected with Leginon on an FEI Tecnai TF20 operating at 200 kV equipped with a Gatan K2 Summit direct detector at a 29,000x magnification, following the procedure previously described [39]. The detector was operated in the counting mode at a dose rate of 9 electrons per pixel per second. Each movie was recorded over 5 seconds and consisted of 25 frames. Movie frame alignment was performed using a frame offset of 7 along with a B factor of 1000 pixels-squared to correct stage shift and beam-induced motion as described [40]. A total of 245 movies were collected, and 88,884 particles were boxed from motion-corrected 25-frame averages with the program DoGPicker with a dimension of 192x192 pixels and a pixel size of 1.21 Å. CTF correction was performed with the program CTFFind [36], with calculated defocus values ranging from -1.65 to -3.90 μm. The boxed particle images were binned by a factor of 2 to 2.42 Å/pixel and used for subsequent 2D classification and image analysis with RELION. No symmetry was applied during the 2D classification.

### Solution X-ray scattering

The purified gp12 monomer was subject to the online SEC small-angle X-ray scattering (SEC-SAXS) at the Bio-SAXS beamline BL4-2 at the Stanford Synchrotron Radiation Lightsource (SSRL) using a Rayonix MX225-HE CCD detector (Rayonix, Evanston, IL) with a sample-to-detector distance of 1.7 m and a beam energy of 11 keV (wavelength λ = 1.127 Å) [41] (S1 Table). Data collection and analysis for the SEC-SAXS were performed as previously described [42]. Briefly, 100 μl protein sample at 5.6 mg/ml was applied onto a Superdex 200 PC 3.2/30 column equilibrated in the protein buffer with 5mM DTT. Eluate from the column was directly passed through a 1.5-mm-quartz capillary cell (Hampton Research, Aliso Viejo, CA) at 20°C in line with the X-ray beam. Scattering images were recorded with 1.5-second exposures every 5 seconds using the program Blu-Ice [43]. The program SasTool (http://ssrl.slac.stanford.edu/~saxs/analysis/sastool.htm) was employed for data reduction including scaling, azimuthal integration, averaging and background subtraction. The first 100 images at the early part of the void volume were averaged and used as a buffer-scattering profile for the background subtraction. The scattering profiles were manually averaged after visual inspection in order to improve the signal-to-noise ratio. Guinier analysis was performed using the programs Primus [44] and AUTORG [45].

## Results

### The phage Sf6 DNA-injection protein gp12 forms a decameric assembly

The phage Sf6 DNA-injection protein gp12 is a 431-residue, 46.5kDa protein. Sequence analysis with TMpred (http://www.ch.embnet.org/software/TMPRED_form.html) predicted two potential transmembrane helices in gp12 encompassing residues 1–18 and 46–64 respectively (S1A Fig). Residue 222 to 293 was predicted to be a coiled-coil region (http://www.ch.embnet.org/software/COILS_form.html) (S1A Fig). We have heterologously expressed and purified gp12. The gp12 existed as insoluble proteins in inclusion bodies when expressed in *E*. *coli*. We utilized a denaturing-and-refolding approach, which yielded soluble proteins showing two peaks on size exclusion chromatography (SEC) corresponding to a monomer and a decamer respectively (S1B Fig), although the ratio of the two peaks varied from batch to batch. When the fractions of the decamer peak were pooled and passed onto an SEC again, only the decamer peak was observed (Fig 1A), suggesting that the decamer was stable and there was no significant dissociation. When the purified monomeric gp12 was incubated after days of storage under 4°C and passed on an SEC again, three peaks were observed, corresponding to a monomer, a potential dimer and a decamer (S1C Fig), suggesting that the gp12 monomer can spontaneously assemble into the decamer.

**Fig 2:**
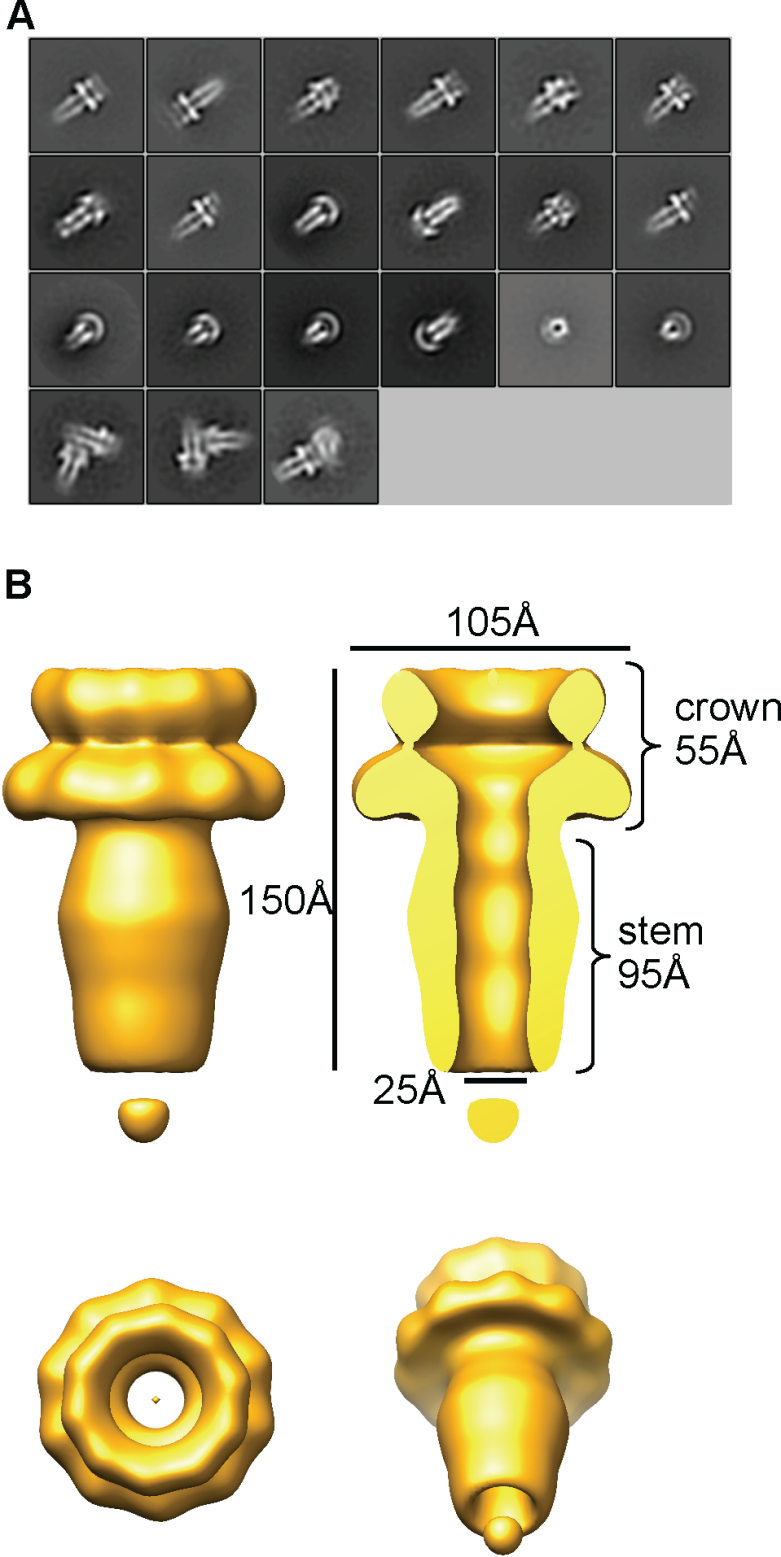
3D reconstruction of the gp12 decameric assembly. (A) Class averages of the negatively stained gp12 decamer. The bottom row shows three class averages corresponding to stacked double decamers, demonstrating robustness of the 2D classification. The box size for class averages is 349.4 Å. (B) 3D reconstruction. Top left, front view. Top right, cutaway view. Bottom left, top view. Bottom right, tilted by 45° from the view on the left.

Negative staining EM of the purified gp12 decamer showed a ring-like structure with a central hole (Fig 1B). We collected electron cryo-microscopy (cryoEM) images of the frozen-hydrated gp12 decamer using a direct electron detector. CryoEM images confirmed the ring-like structure (Fig 1C), and class averages generated by 2D classification with the program RELION [37] clearly showed a 10-fold symmetry (Fig 1D; S2 Fig). However, the 10-fold symmetry was not discernable in class averages from 2D classification of the negative staining images (Fig 2A), presumably due to the staining effect. Moreover, class averages generated from reference-free 2D classification of cryoEM images only showed 10-fold symmetry, but not any other type of symmetries such as 9-, 11-, and 12-fold symmetry. This suggests that the 10-fold symmetry is dominant, and gp12 expressed and purified in vitro has a strong tendency to form the 10-fold symmetric oligomer instead of oligomers with other stoichiometry. We can’t rule out the possibility that other oligomeric states such as 9-, 11- or 12-mers also exist, but such states must represent very small and negligible fractions in the sample. These data suggest that gp12 in vitro is capable of assembling into a ring-like decameric structure.

### The monomeric form of gp12 exhibits high flexibility in solution

Small-angle X-ray scattering (SAXS) shows a characteristic peak in the low *q* region followed by a drop in the high *q* region in the Kratky plot of the gp12 decamer, indicating a well-folded, globular protein complex (S1C Fig). In comparison, the Kratky plot of the gp12 monomer does not have such a characteristic peak but increases monotonically with *q* and displays a plateau at the high *q* region (S1C Fig). For a completely unfolded protein, the Kratky plot increases monotonically with q without a plateau at the high q region. These data suggest that the gp12 monomer is highly flexible and may be partially unfolded. These data suggest that gp12 monomer may exist in solution in a metastable conformational state that is highly flexible and possibly partially unfolded, whereas the decamer possesses a well-defined globular structure.

### The 3D structure of the gp12 decameric assembly

2D classification using negative staining EM images of gp12 decamer resulted in class averages representing various views of the particle, consistent with an overall mushroom-like structure with a cap, a long stem and a channel throughout (Fig 2A). However, cryoEM images dominantly show views approximately down the ring axis (Fig 1C), which suggests preferential orientation and is consistent with the presence of two predicted N-terminal transmembrane helices at the N-terminus (S1A Fig). 2D classification of cryoEM images also showed views tilted by small angles (for example, the last three in the left panel of Fig 1D), indicating an overall architecture that is in agreement with the negative staining EM result.

Because 2D classification using negative staining EM images showed class averages of various orientations including side views, tilted views and top/bottom views (Fig 2A) whereas cryoEM images showed preferential orientation (Fig 1C), we used negative staining images for 3D reconstruction applying the 10-fold symmetry. 3D reconstruction with the negative staining EM images yielded a structure at 13.4 Å resolution using the FSC = 0.5 criterion (Fig 2B; S3 Fig). The assembly shows a ~150-Å, tube-like structure consisting of a crown domain and a stem domain. The ~55 Å tall crown domain is formed by two stacks of ring-shaped density connected by a thin density, with the top and bottom stacks of a diameter of ~85 Å and 105 Å respectively. Those two stacks of rings may correspond to two subdomains in the gp12 protein structure. The stem domain embraces an elongated channel with an inner diameter of 25 Å, which extends to the bottom ring of the crown domain, whereas the inner diameter of the upper ring in the crown domain has a wider inner diameter. The stem domain has an outer diameter ranging from ~50 at the narrowest position to 65 Å at the widest position. In the class averages of cryoEM images (Fig 1D; S2 Fig), each of the ten punctate-like density is ~4 pixels in diameter and ~4 pixels apart, corresponding to 9.48 Å given the pixel size of 2.42 Å. This is reminiscent of the size of an α-helix. While is it tempting to postulate these punctate-like density represent 2D projections of α-helices down the longitudinal axes, a high resolution structure is required to address this.

### The Sf6 gp12 exhibits structural modularity

Sequence analysis shows significantly higher (50%) identity for residues 1–233 but much lower (16%) for remaining residues between Sf6 gp12 and its phage P22 ortholog gp20 (S4 Fig) [31]. Interestingly, sequence analysis identified three phages, Sf6, a Shigella phage Sf101 and a Salmonella phage ST160, whose Sf6 gp12 orthologous proteins show apparent sequence mosaicity (S4 Fig). Residues 1–179 of Sf6 gp12 are essentially identical to the N-terminal portion of the ortholog protein in Sf101 (92% sequence identity), whereas the remaining residues display a much lower identity (33%). Between Sf6 and ST160, residues 1–179 show lower identity (43%) whereas the remaining residues show a significantly higher identity (89%). These data suggest that gp12-class proteins may consist of domains with modular functions that are readily switchable among phages via possible genetic mechanisms such as recombination, which is consistent with the two-domain architecture as shown in the Sf6 gp12 decamer structure.

## Discussion

### The constricted channel in Sf6 gp12 decamer

In this study, we isolated and assembled Sf6 DNA-injection protein gp12 *in vitro*. We showed that gp12 assembled into a ring-like decamer with a crown domain and a stem domain, forming a channel with a length of 150 Å and an inner diameter of 25 Å. It has been known for long that podoviruses such as P22 and T7 encode several proteins that are translocated to the host cells and are essential for DNA injection [9, 11]. However, the exact roles of those DNA-injection proteins have been largely unclear. The channel observed here in the Sf6 gp12 assembly suggests a direct role of gp12 in mediating phage DNA injection, that is, providing a molecular conduit for DNA translocation.

**Fig 3:**
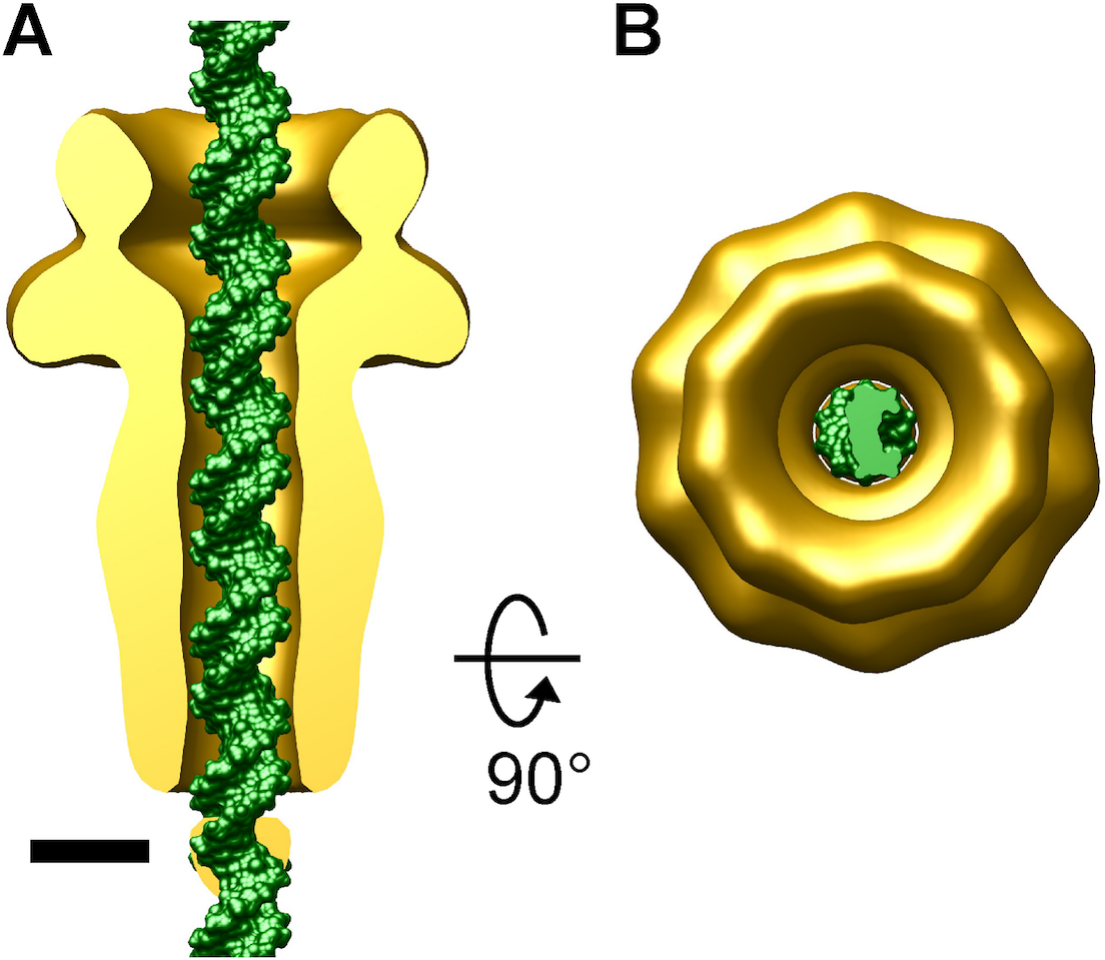
Fitting of dsDNA into the channel of the gp12 decameric assembly. The gp12 structure fitted with a standard dsDNA molecule (green molecular surface). (A), side view. (B), a view down the gp12 channel longitudinal axis. In (A), the front half of the gp12 assembly is computationally removed to show the channel width. Bar, 25 Å.

The gp12 decamer stem domain forms a constricted channel with an inner diameter of 25 Å, which accommodates B-form dsDNA taking into account errors arising from the negative staining artifact and the limited resolution of the 3D reconstruction (Fig 3). The matching between the inner diameter of the gp12 channel and the dsDNA outer diameter indicates that the translocating DNA likely makes tight contact with the gp12 protein. An elongated channel was observed in the phage P22 portal structure [18, 46]. The 25-Å inner diameter of the Sf6 gp12 assembly represents a narrower channel than that observed in P22 portal which is over 30 Å in diameter [46]. Thus, during DNA injection, the Sf6 gp12 channel may closely hold the DNA, and therefore, may play a valve-like regulatory role for DNA translocation. Phage DNA injection was thought to be driven by the DNA self-repulsion and bending energies [30, 47, 48] and/or osmotic pressure gradients [26] due to the highly densely packaged DNA in the capsid. It is likely that the constricted molecular channel in the gp12 assembly is adapted for rapid uni-directional DNA translocation, probably by maximizing the efficiency of translating the driving energy into DNA movement.

The localization of the DNA-injection proteins in phages Sf6 and P22 is not clear. In phage T7, DNA-injection protein gp14 was partitioned to infect cell outer membrane, while gp15 and gp16 were shown to be associated with both outer and inner membrane thus probably spanned the periplasm and the cytoplasmic membrane [10]. Consistent with the preferential orientation on EM grids, it is likely that the two predicted N-terminal transmembrane helices in Sf6 gp12 are located at one end of the 3D structure, which may anchor the assembly to the host cell membrane, though it remains to be addressed if it is anchored to the inner or outer membrane.

The 150-Å length of the Sf6 gp12 assembly may not be long enough to span the entire cell envelope of the Gram-negative host. Indeed, it was shown by electron tomography that phage T7 formed a tail extension with an overall length of ~450 Å traversing the host cell envelope [12]. Presumably, the other two DNA-injection proteins of phage Sf6, gp11 and gp13, may be needed to assemble the complete molecular apparatus that spans the host cell envelope.

### The 10-fold symmetry of the gp12 assembly

**Fig 4:**
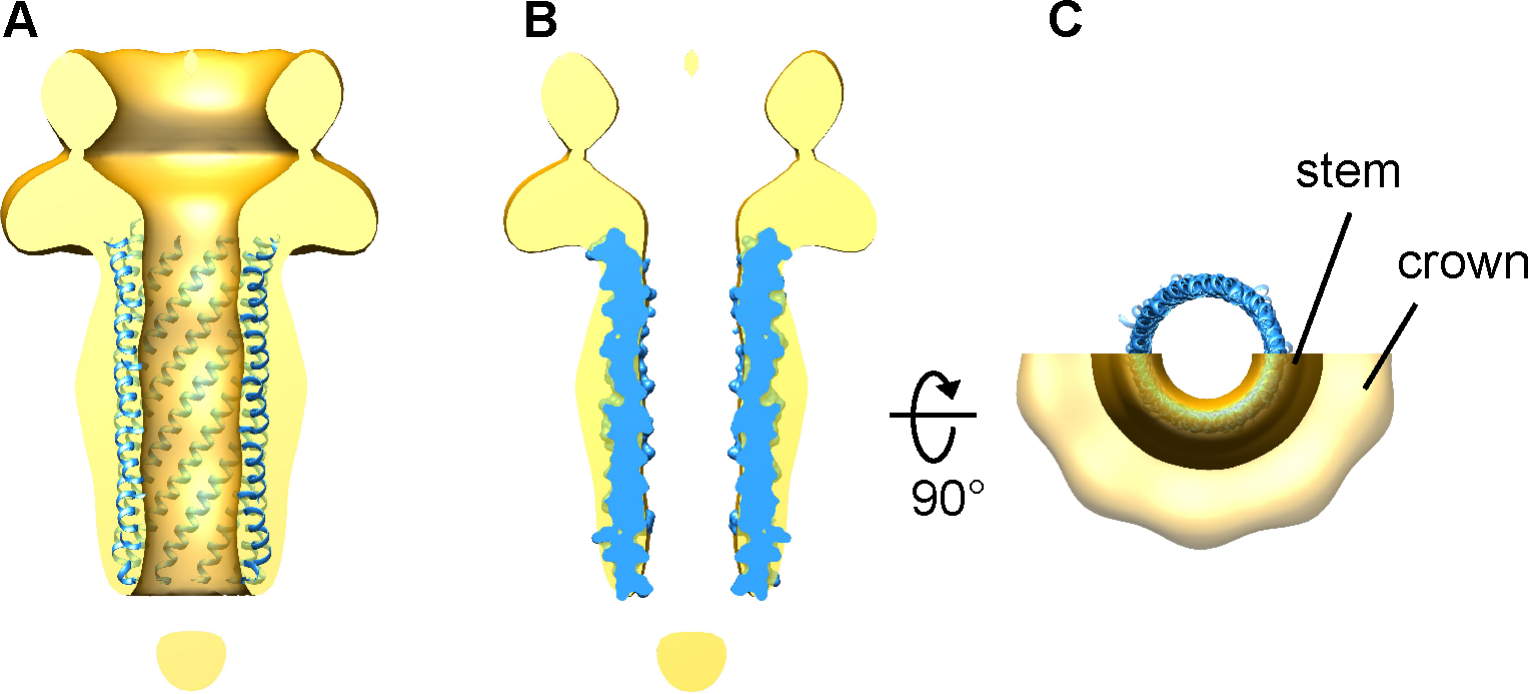
Fitting the phiX174 H protein central domain X-ray structure (blue; RCSB PDB code 4JPP) into the stem domain of the Sf6 gp12 EM map (gold). (A) The H protein structure is shown as ribbon diagram. The front halves of the H structure and the gp12 map were computationally removed for clarity. (B) A corss section of the gp12 map fitted with the H protein structure (molecular surface). (C) A view down the gp12 channel axis. The top half of the gp12 map was removed for clarity. The crown and stem domains of gp12 are indicated.

The Sf6 gp12 assembly shows a 10-fold rotational symmetry. 10-fold symmetry was observed in the X-ray structure of a 140-residue central domain of the H protein of single-stranded DNA (ssDNA) phage phiX174, which showed an elongated tube-like structure that provides a channel throughout the host cell envelope for phage DNA translocation [49]. The X-ray structure of the H protein central domain fits well into the stem domain of the Sf6 gp12 decamer (Fig 4). Sequence analysis predicts a coiled-coil region for residues 223 to 293 in Sf6 gp12 (S1A Fig). CryoEM analysis shows that the gp12 decamer stem domain display features reminiscent of helical elements (Fig 1D). It is well known that juxtaposed alpha-helices form coiled-coil structures, usually in the forms of dimers, trimers and tetramers, but 10-fold symmetric coiled-coil structure was not documented previously [50]. Thus, the X-ray structure of the phiX174 H protein central domain and the EM structure of the full-length Sf6 gp12 assembly reported here may represent first examples of molecular assemblies formed by coiled coils that display 10-fold rotational symmetry to the best of our knowledge. Sf6 is a member of tailed dsDNA phages with a *T* = 7 icosahedral capsid of ~650 Å in diameter, whereas phiX174 has a circular ssDNA genome, is tailless, and has a small *T* = 1 icosahedral capsid of ~260Å. Hence, the 10-fold symmetry may be a common feature for DNA-injection proteins in P22-like bacteriophages as well as tailless ssDNA phages such as phiX174, suggesting a converged adaptation to the rapid DNA translocation function in these phages. In comparison, three DNA-injection proteins in phage T7, gp14, gp15 and gp16, are present in 12, 8 and 4 copies in the virion, respectively, and it is not known what symmetry these proteins assume in the assembled tail extension, which could differ from Sf6 gp12.

It is important to point out that the gp12 decamer in the present study was prepared in vitro and assembled from recombinantly expressed and refolded protein. It is possible that in vitro expressed and purified protein may assemble with a stoichiometry different from the physiological state. For example, the bacteriophage SPP1 portal protein purified in vitro showed 13-fold instead of the physiological 12-fold symmetry [51]. The in vitro purified portal protein from phage T4 [52] showed multiple stoichiometries such as 11-, 12- and 13-fold symmetry, although the physiological 12-fold symmetric oligomer was dominant. Thus, it can’t be ruled out that gp12 may assume a different oligomeric state in its physiological environment. However, our analysis demonstrates that the 10-fold symmetry is dominant, suggesting that gp12 has a strong tendency to form the decamer instead of other oligomeric states. Moreover, the DNA-injection proteins must be translocated from phage interior to the host cell envelope to assemble into the DNA-injection apparatus, which presumably requires at least partial unfolding and refolding of proteins in order for those protein molecules to pass through the channel in the portal complex and the tail. The denaturing and refolding process for gp12 assembly used in the present study may mimic such a physiological process, supporting the notion that the observed 10-fold symmetry in vitro might represent the physiological state. Further studies are needed to verify the physiological stoichiometry by, for example, isolating gp12 from its native environment.

### The decameric gp12 structure suggests a symmetry mismatch with respect to the phage tail

The DNA-injection proteins must attach to the distal end of phage tails to form an extensible channel for DNA injection across the host bacterial envelope. The cryoEM structures of phage P22 and its isolated tail assembly showed 6-fold rotational symmetry at the distal end of the phage tail occupied by the phage protein gp10 [16–19], and such 6-fold symmetry is also true for phage Sf6 tail [23]. While the 10-fold symmetry observed here is for gp12 assembled in vitro, the oligomeric state of gp12 in the physiological environment may be a decamer or at least a stoichiometry close to the decamer, and it is less likely to adopt a much lower-order oligomer such as a hexamer. Thus, the oligomeric structure of gp12 likely suggests a new symmetry mismatch between the DNA-injection proteins and the phage tail, even though it is not known if gp12 attaches to the phage tail through direct or indirect molecular interaction. What is the advantage to have such a symmetry mismatch? It is well known for tailed dsDNA phages that there is symmetry mismatch between the 12-fold symmetric portal protein complex and a 5-fold vertex of the icosahedral capsid where the portal is embedded [53]. It was thought that such symmetry mismatch might reduce the energy barrier for rotation of the two mismatched rings, thus facilitating rotation of the portal complex with respect to the capsid during DNA packaging into phage capsid assuming that the portal acts as a nut through which the DNA screws into the capsid [54]. Although a previous single molecule study of phage phi29 showed that such rotation of the portal complex with respect to the capsid might not be absolutely necessary [55], a more recent study showed that the phi29 DNA-packaging ATPase drove the rotation of DNA during packaging, thus supported the DNA rotation with respect to the portal [56]. The ~25 Å inner diameter of the Sf6 gp12 channel suggests tight contact between DNA and the amino acid residues lining the inner wall of the gp12 channel, thus the gp12 might act like a nut through which the translocating double helical DNA passes. Due to the helical nature of the dsDNA, this may result in rotation of the gp12 with respect to the DNA. The symmetry mismatch between the gp12 assembly and the 6-fold phage tail is consistent with such a potential mechanism, that is, gp12 may be rotating during DNA translocation, by reducing the energy barrier for rotation. Thus, it would be interesting to perform further studies to test if such a rotation indeed occurs during DNA injection.

### Implications for translocation of gp12 and assembly at the host cell envelope

**Fig 5:**
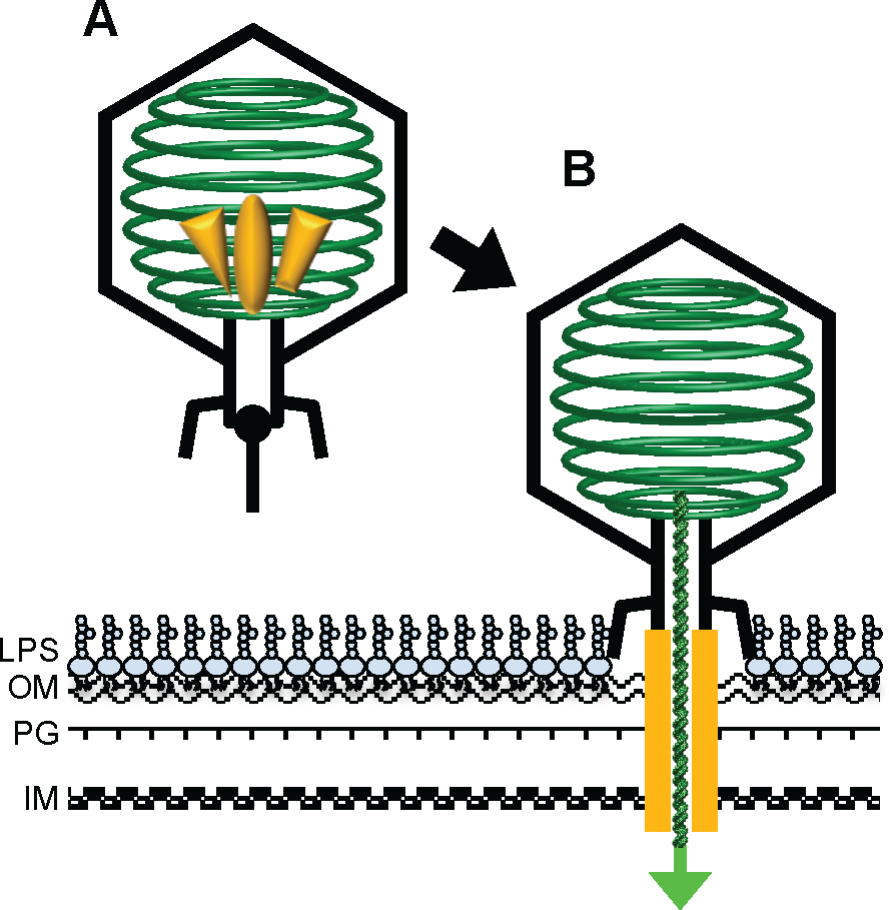
Schematic representation of translocation of the DNA-injection proteins and the assembly of the DNA-injection device in the host envelope. (A) The three DNA-injection proteins, gp12, gp11 and gp13, represented with an oval, triangle and trapezoid in blue, are packed in the phage particle. Note that the copy numbers of those proteins in the capsid and how they are arranged are not known. (B) upon attachment to the host cell, the DNA-injection proteins are translocated through the tail channel to the host cell envelope, where they assemble into an extended tube-like structure to allow delivery of phage DNA into the host cytoplasm. The arrangement of those proteins is not known and is drawn schematically. LPS, lipopolysaccharide; OM, outer membrane; PG, peptidoglycan; IM, inner membrane. The phage dsDNA is in green.

DNA-injection proteins are structural proteins that are packed in mature phage particles, forming well-defined core in phages such as T7 [57, 58] or being not structurally ordered in phages such as P22 [18]. It remains unclear how these proteins are translocated to the host cell surface where they assemble and function, but it is highly likely that these proteins are translocated through the portal and tail channel, which is the only conduit connecting phage interior to exterior. Our SAXS data show that gp12 monomer exhibits high flexibility in solution and may be partially folded, that is, part of the protein is folded, but the remaining portion, for example, those alpha-helical elements that make up the tube structure, may have already formed secondary structures but may remain flexible before being packed into the well define 3D structure in the decamer. Given the elongated tube-like structure of the gp12 decameric assembly, the gp12 monomer likely exists as a flexible, extended, slender molecule in solution. Such a slender conformational state might enable gp12 to translocate from the interior of the phage particle through the conduit formed by the portal and the tail, or even through the crevice between the DNA and the proteins forming the portal and tail, since the portal/tail conduit may have already been occupied by the leading end of the DNA to be injected as shown in phage P22 [18]. Upon arrival at the host cell envelope, it assembles into the decameric complex to provide a molecular channel for translocation of phage DNA into the host bacterial cytoplasm while protecting the DNA from digestive enzymes in the host periplasmic space (Fig 5). Our structural results shed light on such an elegant, assembled-on-site mechanism for a molecular apparatus that injects DNA across multiple physical and chemical barriers in the Gram-negative host cell envelope. Such a mechanism may be common among many dsDNA phages such as P22-like and T7-like phages as well as ssDNA phages such as phiX174. Further studies are under way for collection of new cryoEM data by using an appropriate detergent or a supporting film to eliminate the issue of preferential orientation, which is expected to generate a higher resolution cryoEM structure.

## Supporting Information

S1 Fig Biochemical analysis of gp12. (A) A schematic of the gp12 domain organization. Transmembrane helices and the coiled-coil region were predicted with TMpred (http://www.ch.embnet.org/software/TMPRED_form.html) and COILS (http://www.ch.embnet.org/software/COILS_form.html) respectively. (B) Size-exclusion chromatography (SEC) of gp12 showing peaks corresponding to the monomer and the decamer respectively. The right panel shows SDS-PAGE of the fractions of the decamer peak (Lanes 1–4) and monomer peak (Lanes 5–8). The gp12 position is indicated with an arrow. MW, molecular weight marker. (C) SAXS of gp12. The left panel shows three peaks in SEC elution profile (blue curve) immediately prior to SAXS data collection, corresponding to the decamer, a potential dimer and the monomer respectively. The right panel shows the normalized Kratky plots of the three peaks in SEC elution profile, indicating that the species in Peak 1 is well folded whereas those of Peak 2 and 3 are rather flexible.
(TIFF)

S2 Fig Enlarged views of the two class averages of cryoEM images of the purified gp12 decamer as shown in Fig 1D right panel. Each pixel is clearly seen as a square so that the number of pixels can be counted for each punctate-like density (red arrows), which is 4 pixel or 9.48 Å given the 2.42 Å pixel size, fitting well with an alpha-helix.
(TIFF)

S3 Fig A plot of the Fourier shell correlation with respect to the spatial frequency. (TIFF)

S4 Fig Alignment of Sf6 gp12 sequence with that of its homolous protein in phages P22 (A), Sf101 (B) and ST160 (C) respectively. The sequence alignment was performed with Clustal omega (http://www.clustal.org) and the figure was generated with ESPript (http://espript.ibcp.fr/ESPript/). Identical residues are shown in white letters in the red background. Similar residues are shown in red letters in a white background.
(TIFF)

S1 Table Data collection and structural parameters for small-angle X-ray scattering. (DOCX)
