# Supplementary figures and images for: Structure of a Bacterial Virus DNA-Injection Protein Complex Reveals a Decameric Assembly with a Constricted Molecular Channel
Source: PLoS One. 2016 Feb 16;11(2):e0149337. doi: 10.1371/journal.pone.0149337 (PMC4755594; doi:10.1371/journal.pone.0149337)

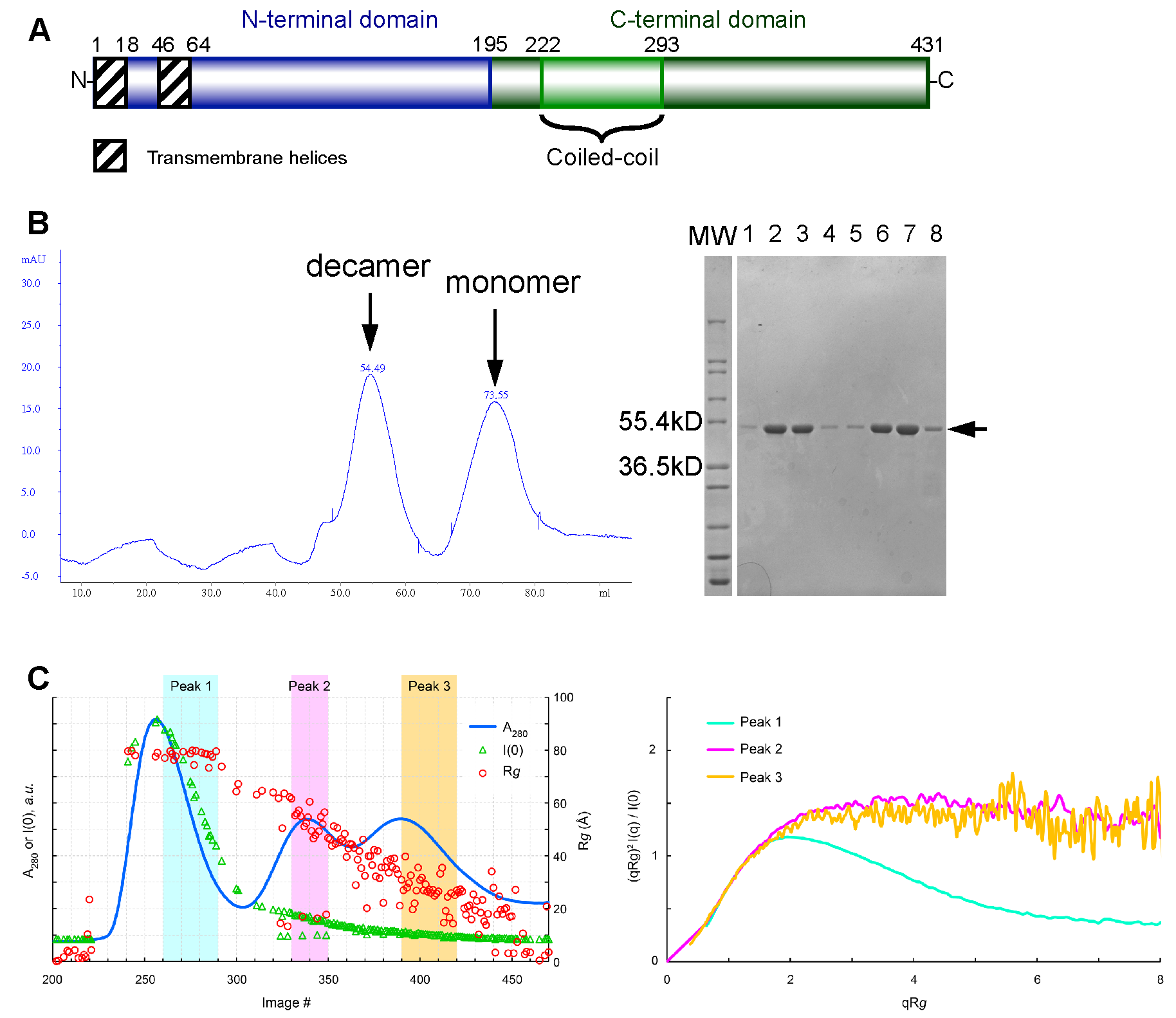

Supplement: S1 Fig — (A) A schematic of the gp12 domain organization. Transmembrane helices and the coiled-coil region were predicted with TMpred (http://www.ch.embnet.org/software/TMPRED_form.html) and COILS (http://www.ch.embnet.org/software/COILS_form.html) respectively. (B) Size-exclusion chromatography (SEC) of gp12 showing peaks corresponding to the monomer and the decamer respectively. The right panel shows SDS-PAGE of the fractions of the decamer peak (Lanes 1–4) and monomer peak (Lanes 5–8). The gp12 position is indicated with an arrow. MW, molecular weight marker. (C) SAXS of gp12. The left panel shows three peaks in SEC elution profile (blue curve) immediately prior to SAXS data collection, corresponding to the decamer, a potential dimer and the monomer respectively. The right panel shows the normalized Kratky plots of the three peaks in SEC elution profile, indicating that the species in Peak 1 is well folded whereas those of Peak 2 and 3 are rather flexible. (TIFF) [file pone.0149337.s001.tiff]

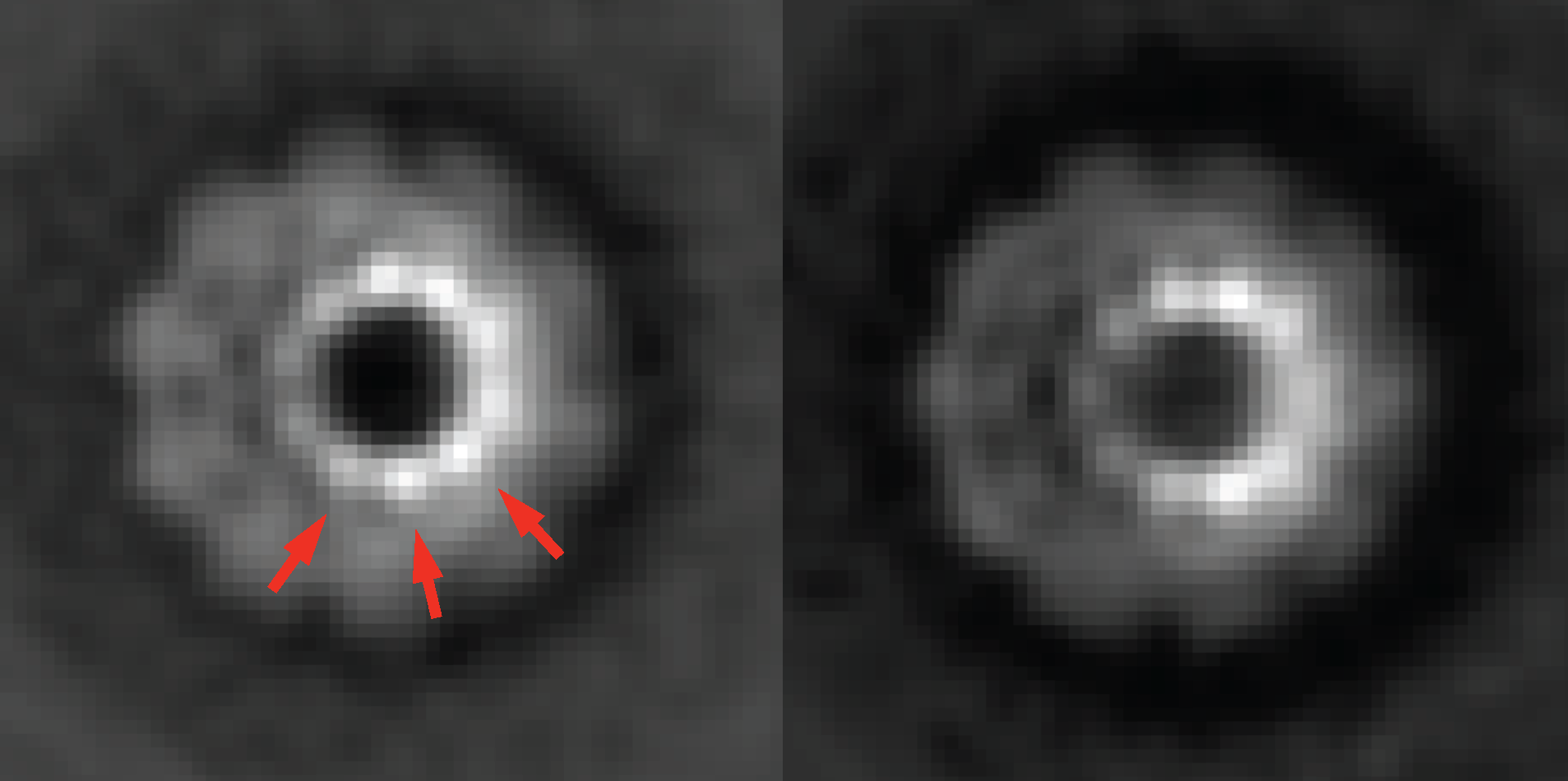

Supplement: S2 Fig — Each pixel is clearly seen as a square so that the number of pixels can be counted for each punctate-like density (red arrows), which is 4 pixel or 9.48 Å given the 2.42 Å pixel size, fitting well with an alpha-helix. (TIFF) [file pone.0149337.s002.tiff]

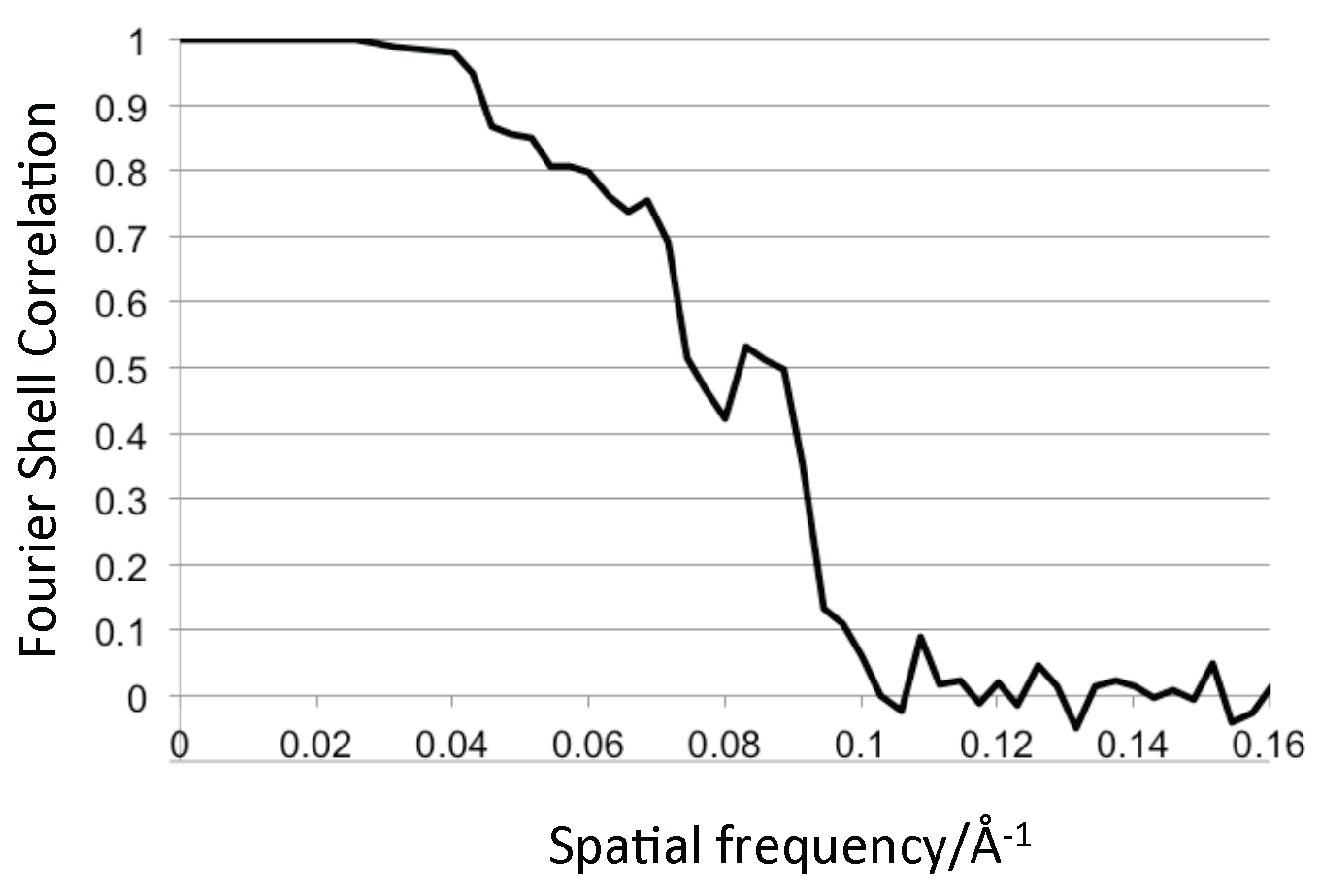

Supplement: S3 Fig — (TIFF) [file pone.0149337.s003.tiff]

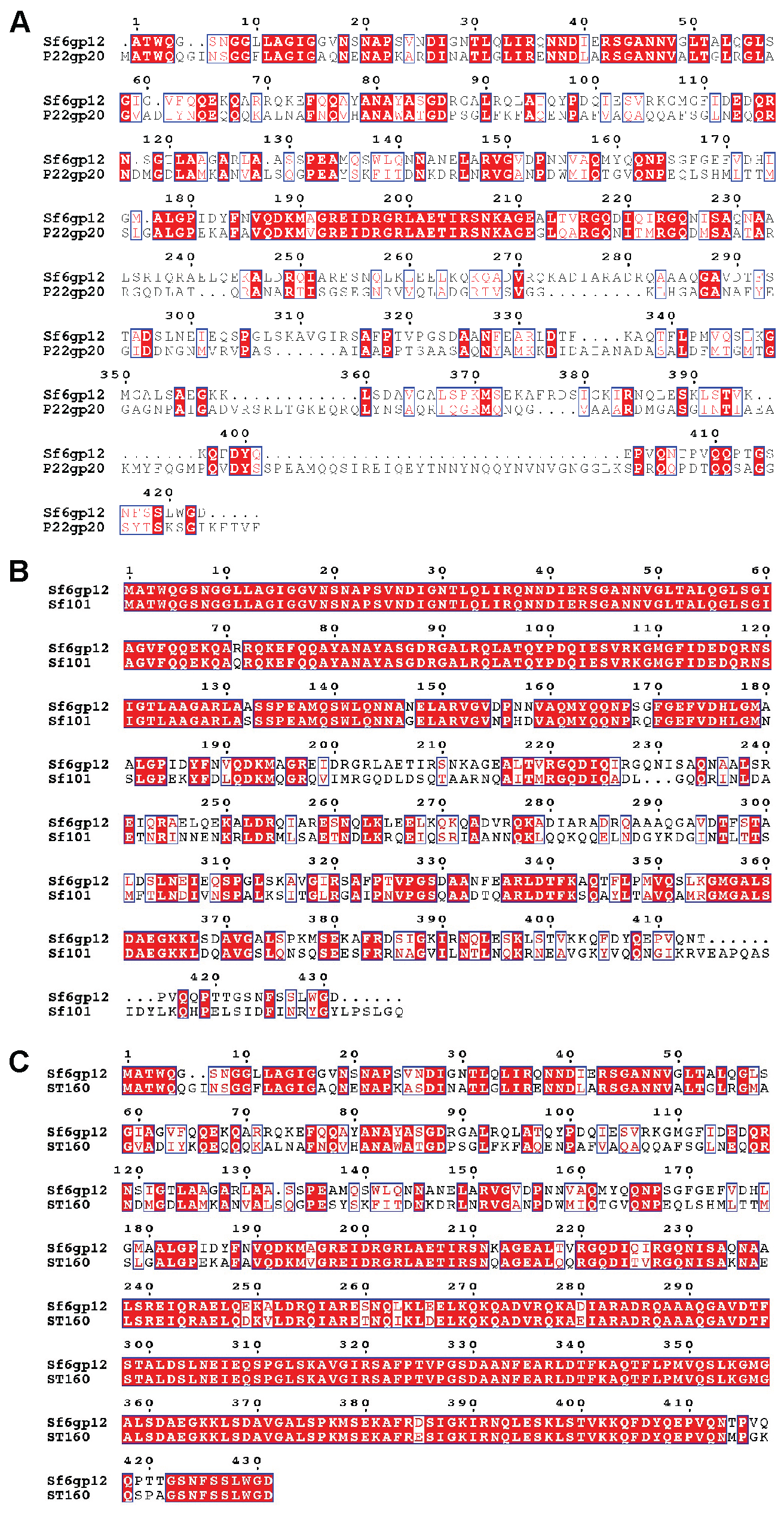

Supplement: S4 Fig — Alignment of Sf6 gp12 sequence with that of its homolous protein in phages P22 (A), Sf101 (B) and ST160 (C) respectively. The sequence alignment was performed with Clustal omega (http://www.clustal.org) and the figure was generated with ESPript (http://espript.ibcp.fr/ESPript/). Identical residues are shown in white letters in the red background. Similar residues are shown in red letters in a white background. (TIFF) [file pone.0149337.s004.tiff]
