# Supplementary material for: Structure of a Bacterial Virus DNA-Injection Protein Complex Reveals a Decameric Assembly with a Constricted Molecular Channel
Source: PLoS One. 2016 Feb 16;11(2):e0149337. doi: 10.1371/journal.pone.0149337 (PMC4755594; doi:10.1371/journal.pone.0149337)
Supplement: S1 Table — (DOCX) [file pone.0149337.s005.docx]

S1 Table. Data collection and structural parameters for small-angle X-ray scattering.

| **Data Collection Parameters** |  |
| --- | --- |
| Instrument | SSRL BL4-2 |
| Detector distance (m) | 1.7 |
| Beam energy (keV), Wavelength (Å) | 11, 1.127 |
| Beam current (A) | 600 |
| Defining slits size (horizontal/mm x vertical/mm) | 0.3 x 0.3 |
| Q range (Å^-1^) | 0.008-0.46 |
| Size of quartz capillary in diameter (mm) | 1.5 |
| Exposure time per frame (s) | 1.5 |
| Frame per FPLC experiment | 600 |
| Temperature (K) | 293 |
| SEC column | Superdex 200PC 3.2/30 |
| FPLC flow rate (ml/min) | 0.05 |
| Sample volume applied (ul) | 100 |
| Sample concentration (mg/ml) | 5.6 |
| **Structural Parameters** |  |
| Image frames used for analysis | Peak 1: 260-289  Peak 2: 330-349  Peak 3: 390-419 |
| I(0) from Guinier analysis | Peak 1: 4995.0 (+/- 63.2)  Peak 2: 646.78 (+/- 19.1)  Peak 3: 167.20 (+/- 2.09) |
| Rg (Å) from Guinier analysis | Peak 1: 79.5 (+/- 1.09)  Peak 2: 51.7 (+/- 1.76)  Peak 3: 31.7 (+/- 0.57) |
